# Adiponectin Inhibits AURKA to Suppress Inflammation in TNF‐α‐induced Keratinocytes and Attenuates Psoriatic Dermatitis in Mice

**DOI:** 10.1002/iid3.70407

**Published:** 2026-03-23

**Authors:** Lingling Zhang, Chunxi Ke, Yun Shen, Feng Shi, Qingqing Jiao, Jiang Ji

**Affiliations:** ^1^ Department of Dermatology The Second Affiliated Hospital of Soochow University Suzhou China; ^2^ Department of Dermatology Gongli Hospital of Shanghai Pudong New Area Shanghai China; ^3^ Department of Dermatology Suzhou Municipal Hospital Suzhou China; ^4^ Central Research laboratory The First Affiliated Hospital of Soochow University Suzhou China; ^5^ Department of Dermatology The First Affiliated Hospital of Soochow University Suzhou China

**Keywords:** adiponectin, Aurora kinase A, Forkhead transcription factor 1, psoriasis, TNF‐α

## Abstract

**Introduction:**

Psoriasis is a recurrent immune‐mediated systemic disease. Adiponectin (APN), a key regulator of metabolism, is also known for its anti‐inflammatory properties in several inflammatory disorders. The study aims to investigate the anti‐inflammatory properties of APN on human immortalized keratinocyte cells (HaCaT) and to evaluate its therapeutic potential in an imiquimod (IMQ)‐induced psoriasis mouse model.

**Methods:**

HaCaT cells were treated with 5, 10, or 20 μg/ml APN, and cell viability was assessed. A psoriasis‐like cellular model was created by exposing HaCaT cells to TNF‐α (50 ng/ml) for a duration of 24 h. Apoptosis was analyzed using flow cytometry, and the secretion of inflammatory cytokines was measured through enzyme‐linked immunosorbent assay (ELISA). Real‐time quantitative polymerase chain reaction (RT‐qPCR) was used to measure the mRNA expression levels of AdipoR1, AdipoR2, and T‐cadherin(T‐cad). Aurora kinase A (AURKA) and Forkhead transcription factor 1 (FOXM1) were analyzed using Western blotting (WB) and RT‐qPCR. The anti‐psoriatic effect of APN was also evaluated in IMQ‐induced psoriatic dermatitis. Additionally, ELISA and WB were used to assess cytokines and key signaling proteins in mouse skin tissues.

**Results:**

APN significantly inhibited the proliferation of HaCaT cells and enhanced their apoptosis. Additionally, it decreased the production of interleukin (IL)‐1β, IL‐8, and IL‐6. APN upregulated AdipoR1 and AdipoR2 mRNA levels while downregulating the mRNA and protein levels of T‐cad. Mechanistically, APN mitigated the inflammatory response in keratinocytes by suppressing the TNF‐α‐induced upregulation of AURKA and FOXM1. This mechanism was substantiated in vivo, where APN treatment alleviated IMQ‐induced psoriatic dermatitis in mice, concurrently reducing levels of IL‐1β, CXCL2 and IL‐6, and modulating the expression of AdipoR1, AdipoR2, AURKA, and FOXM1 in mouse skin.

**Conclusion:**

Our findings suggest that APN inhibits keratinocyte hyperproliferation and suppresses inflammation in TNF‐α‐induced keratinocytes. Moreover, APN treatment attenuates IMQ‐induced psoriatic dermatitis in mice, supporting its potential as a therapeutic approach for psoriasis.

**Abbreviations**
AdipoR1: adiponectin receptor 1
AdipoR2: adiponectin receptor 2
AIM2: absent in melanoma 2
APN: adiponectin
AURKA: aurora kinase A
CCK‐8: cell counting kit‐8
ELISA: enzyme‐linked immunosorbent assay
FOXM1: forkhead transcription factor 1
HaCaT: human immortalized keratinocyte cells
IL: interleukin
IMQ: imiquimod
RT‐qPCR: real‐time quantitative polymerase chain reaction
TNF‐α: tumor necrosis factor alpha
T‐cad: T‐cadherin
WB: Western blotting

## 1 Introduction

Psoriasis, a recurrent and immune‐mediated systemic disease, is characterized by the presence of erythematous plaques and scaling in skin. It affects roughly 2 to 3 percent of the worldwide population and adversely affects patients' social functioning and quality of life [1, 2]. Topical therapies are commonly used as standard treatments for managing mild to moderate psoriasis [3], while biologics and small molecule inhibitors are used for severe cases [4]. However, high treatment costs, side effects, and recurrence rates associated with these treatments underscore the urgent need for more effective treatment alternatives. The pathogenesis of psoriasis is driven by immune cell infiltration, abnormal keratinocyte proliferation, and an imbalanced cytokine environment, especially involving interleukin‐17 (IL‐17) and tumor necrosis factor‐alpha (TNF‐α) [5, 6, 7]. Despite significant progress, the molecular pathways underlying keratinocyte hyperproliferation and inflammation remain incompletely understood.

**Figure 1:**
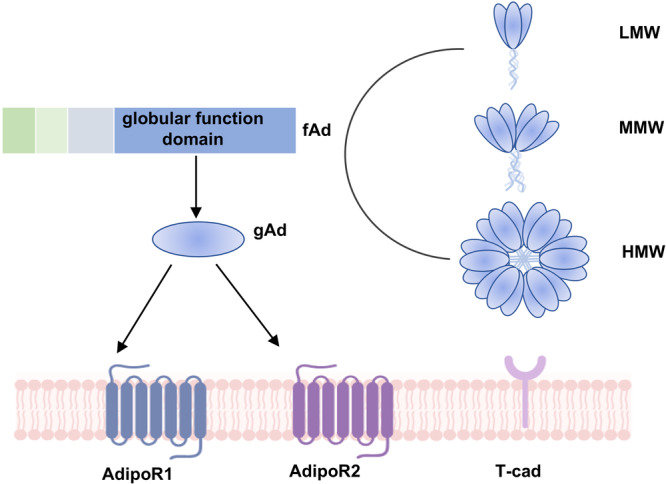
Structural composition and receptor binding mechanism of APN. Adiponectin (APN) is a 30 kDa protein secreted by adipocytes. The globular function domain, also known as globular adiponectin (gAd), is created by breaking down the complete adiponectin (fAd) molecule. There are three types of adiponectin oligomers: high molecular weight (HMW), medium molecular weight (MMW), and low molecular weight (LMW). Adiponectin has three receptors: AdipoR1, AdipoR2, and T‐cad. fAd and gAd both bind to and activate AdipoR1 and AdipoR2. T‐cad only recognizes MMW and HMW oligomers.

Adiponectin (APN), a hormone generated from white adipose tissue, plays a pivotal role in immune regulation and metabolic diseases [8, 9]. Recent observational research has identified a strong correlation between reduced plasma adiponectin levels and a heightened risk of developing psoriasis [10]. Animal model studies have demonstrated that mice lacking adiponectin experience more severe psoriasis‐like skin inflammation, likely due to the potent anti‐inflammatory properties of APN [11]. APN exists in multiple isoforms, including low‐molecular‐weight (LMW), middle‐molecular‐weight (MMW), and high ‐molecular‐weight (HMW) forms [12], which bind to three specific receptors: T‐cadherin (T‐cad), adiponectin receptor 1 (AdipoR1), and adiponectin receptor 2 (AdipoR2). Specifically, AdipoR1 and AdipoR2 can bind to globular APN (gAd), and upon binding, adiponectin activates a series of tissue‐specific signaling pathways, including the upregulation of peroxisome proliferator‐activated receptor α (PPARα) ligand activity tand he activation of adenosine monophosphate‐activated protein kinase (AMPK), which together contribute to the reduction of inflammation in various cell types [13]. In contrast, T‐cad, a special cadherin missing transmembrane and cytosolic domains, has specifical affinity for MMW and HMW but not for gAd or LMW APN (Figure 1) [14].

Clinical studies have demonstrated that serum and lesional tissue levels of APN are markedly decreased in patients with psoriasis [15, 16], and serum APN levels rise after treatment [17]. Studies have shown that APN inhibits the differentiation of Th0 cells through AMPK‐dependent pathways, thereby reducing the production of IL‐17A [18], or inhibits the synthesis and secretion of IL‐17, thus playing a beneficial role in psoriasis [19]. However, our understanding of other cell types affected by APN and their molecular pathways and signal transduction mechanisms remains limited. Most importantly, the specific effects of APN on keratinocytes require further in‐depth exploration, which will provide a more solid scientific support and a strong theoretical basis for the pharmacological treatment of psoriasis.

As previously discussed, the downstream signaling pathways of adiponectin receptors involve AMPK, p38 mitogen‐activated protein kinase (MAPK), and PPARα. Forkhead box protein M1 (FOXM1), as a key downstream effector of the p38‐MAPK‐MK2 and PI3K‐AKT signaling cascades, has piqued our interest [20]. FOXM1 is involved in the regulation of both cell proliferation and inflammatory processes in psoriasis [21, 22]. Notably, Aurora kinase A (AURKA), well‐known for its role in mitosis and tumor development, is upregulated in psoriatic lesions and downregulated after treatment with TNF‐α antagonists [23]. Recent studies further indicate that AURKA suppresses autophagy in keratinocytes to enhance pro‑inflammatory Absent in Melanoma 2 (AIM2) inflammasome activation and promotes keratinocyte proliferation and migration, thereby contributing to psoriatic pathogenesis [24, 25]. Both FOXM1 and AURKA are overexpressed cell cycle‐related genes that engage in a positive feedback loop; FOXM1 can recruit AURKA as a co‐factor to enhance the transcriptional activation of its target genes, utilizing a mechanism independent of AURKA's kinase activity, and FOXM1 directly interacts with the promoter region of the AURKA gene, thereby enhancing its transcriptional activity. This interplay results in the co‐elevation of AURKA and FOXM1 expression levels [20]. However, the relationship between AURKA and FOXM1 in psoriasis, and their combined role in keratinocyte proliferation and inflammation, remains unexplored.

Although previous studies have identified APN as a regulator of psoriasis activity, the specific signaling pathways involved remain unknown. Recent studies have revealed the critical involvement of AURKA and FOXM1 in keratinocyte hyperproliferation, autophagy suppression, and inflammatory activation in psoriasis. However, whether they function as coordinated mediators in adiponectin‑driven regulation remains unclear. To address these knowledge gaps, we aim to explore the impact of APN on keratinocyte proliferation and inflammation, with a focus on the AURKA/FOXM1 signaling pathway. Using a TNF‐α‐stimulated HaCaT cell model, we examined the impact of APN on inflammatory cytokine secretion, the expression of its receptors, and the regulation of AURKA and FOXM1. To further validate the in vitro findings, we assessed whether APN exerts comparable anti‐inflammatory effects in vivo. Specifically, we evaluated the expression of inflammatory cytokines and signaling molecules in the skin of imiquimod‐induced psoriatic mice.

## 2 Materials and Methods

### 2.1 Cellular Components and Reagents

HaCaT cells were kindly provided by the Shanghai Skin Disease Hospital (Shanghai, China). Medium used for cells culture included 10% fetal bovine serum (FBS, YEASEN, China). HaCaT cells were maintained at 37°C in a standard incubator with 5% CO2. The psoriasis model group (TNF‐α group) consisted of HaCaT cells treated with recombinant TNF‐α (50 ng/ml, PeproTech, USA) for a duration of 24 h. Recombinant human (rh) APN was purchased from MCE (USA). MLN8237 (MCE, USA) was used at a final concentration of 0.2 μM to inhibit AURKA activity in HaCaT cells. The cells were then incubated with APN (5, 10, 20 μg/ml) or MLN8237. In subsequent cell‐based assays, untreated cells served as the negative control and TNF‐α‐stimulated cells as the positive model; APN groups were compared with the TNF‐α group unless noted.

### 2.2 Cell Vitality Assay

After seeding HaCaT cells into 96‐well plates, they were exposed to APN (5, 10, and 20 μg/ml, respectively) for either 24 or 48 h. After incubating the plate for 2 h and adding 10 μL of CCK‐8 (MCE) to each well, we assessed the absorbance of each well at 450 nm. In this assay, untreated HaCaT cells served as the control for comparison with APN‐treated groups.

### 2.3 Flow Cytometry to Assess Cell Apoptosis

HaCaT cell apoptosis was evaluated using the Annexin V‐FITC Apoptosis Detection Kit (C1062S, Beyotime). After a 24h APN treatment, 2 × 10⁵ HaCaT cells were resuspended in 200 μL of Annexin V‐FITC buffer. Following this, 5 μL of Annexin V‐FITC and 10 μL of PI were mixed into the suspension.

### 2.4 Enzyme‐Linked Immunosorbent Assay (ELISA)

The supernatant from the cells were preserved at – 80°C until analyzed. ELISA kits from Yubi Trading Co. Ltd. in Shanghai were utilized to measure the concentrations of IL‐6, IL‐1β, and IL‐8 cytokines in cell culture supernatant following the guidelines from manufacturer. To summarize, 100 µl of the culture supernatant was placed into a 96‐well plate, and then various reagents were added. The optical density (OD) was determined at a wavelength of 450 nm.

### 2.5 Real‐Time Quantitative Reverse Transcription‐Pcr (RT‐qPCR)

The total RNA was extracted from HaCaT cells utilizing Trizol reagent (Beyotime). The NovoScript Plus cDNA Synthesis SuperMixes kit from Novoprotein Scientific Inc. in China was employed for cDNA synthesis, SYBR qPCR SuperMix Plus kit was used for RT‐qPCR. The primers listed in Table 1 were obtained from Sangon Biotech Co. Ltd. in Shanghai. Thermal cycling settings include 40 cycles of denaturing at 95°C for 20 s and cooling at 60°C for 1 min after an initial denaturation of 1 min at 95°C. After normalizing to GAPDH, the target genes' relative expression was quantified using the 2^−ΔΔCt^ method.

**Table 1:** Primer sequences Used for RT‐qPCR.

Gene	Primer type	Sequence (5′→3′)
AdiopR1	Sense	AATTCCTGAGCGCTTCTTTCCT
AdiopR1	Antisense	CATAGAAGTGGACAAAGGCTGC
AdiopR2	Sense	TGCAGCCATTATAGTCTCCCAG
AdiopR2	Antisense	GAATGATTCCACTCAGGCCTAG
T‐cad	Sense	ACTAAAGGTTTTAACCGCAC
T‐cad	Antisense	ACTTCCTACAGAAGGCTAAC
AURKA	Sense	GGAATATGCACCACTTGGAACA
AURKA	Antisense	TAAGACAGGGCATTTGCCAAT
FOXM1	Sense	ACCGCTACTTGACATTGGAC
FOXM1	Antisense	GGGAGTTCGGTTTTGATGGTC
GAPDH	Sense	CATGAGAAGTATGACAACAGCCT
GAPDH	Antisense	AGTCCTTCCACGATACCAAAGT

### 2.6 Western Blotting (WB)

HaCaT cells subjected to the designated treatments were lysed using RIPA lysis buffer (Beyotime), and the protein concentration was measured with a BCA protein assay kit (Beyotime). The extracted proteins were separated via SDS‐PAGE and subsequently transferred onto 0.45 μm PVDF membranes under a constant current of 300 mA for 1.5 h. The membranes were then blocked at room temperature for 15 min using a protein‐free rapid blocking buffer (EpiZyme, China). Antibodies targeting AURKA (product code: CST14475, dilution: 1:1000), p‐AURKA(CST2914, 1:1000), FOXM1 (CST5436, 1:1000), T‐cad (ab167407, 1:1000), and GAPDH (60004‐1‐Ig, 1:50000) were procured from Cell Signaling Technology (Beverly, MA), Abcam (Cambridge, MA), and Proteintech (Wuhan, China), respectively.

Samples were blocked at 4°C with th e primary antibody for a whole night, the secondary HRP antibody (CST91196, CST7074, 1:3000) acquired from Abcam was applied to block membrane. Using enhanced chemiluminescence (ECL) kits from EpiZyme, protein bands are observed. NIH image J software was applied to analysis protein bands.

### 2.7 IMQ‐Induced Psoriatic Dermatitis

Female BALB/c mice had unrestricted access to food and were housed in certain circumstances designed to eliminate pathogens. At random, 24 mice were split into 4 groups (*n* = 8): imiquimod (IMQ), control, IMQ+low‐dose APN, IMQ+high‐dose APN. their dorsal hair was shaved at a surface area of 2 × 3 cm^2^. To produce an IMQ‐induced psoriatic dermatitis, a topical dose of 62.5 mg 5% IMQ cream was given once a day for 7 days. In the control group, mice were given 62.5 mg petroleum jelly ointment. Mice in IMQ+high‐dose APN group and IMQ+low‐dose APN group received intraperitoneal injections of APN at 20 μg and 10 μg per day per mouse, respectively. The IMQ and control groups received the same volume of normal saline treatment. In the animal study, Vaseline‐treated mice served as the negative control and IMQ‐treated mice as the positive psoriatic model, with APN‐treated groups compared against the IMQ model.

#### 2.7.1 PASI Scoring and Histological Assessment

The clinical Psoriasis Area and Severity Index (PASI) was used for daily assessments of the severity of skin lesions. On a scale of 0 to 4, erythema, scaling, and thickening were assigned separate scores: 0 indicates none, 1 signifies minor, 2 represents moderate, 3 denotes marked, and 4 corresponds to extremely marked. Following a 7‐day treatment period, the dorsal skin lesions of mice were removed for detailed histopathological evaluation.

#### 2.7.2 Cytokine Detection in Mouse Skin by ELISA

To assess local inflammation in vivo, skin tissues were homogenized in PBS containing protease inhibitors and centrifuged at 10,000*g* for 10 min at 4°C. The supernatants were collected, and the levels of IL‐1β, CXCL2, and IL‐6 were measured using ELISA kits (Yubi Trading Co. Ltd., Shanghai, China), following the manufacturer's instructions.

#### 2.7.3 WB Analysis of Mouse Skin

After euthanasia on day 7, dorsal skin tissues from each group were harvested, immediately frozen in liquid nitrogen, and stored at −80°C. The samples were homogenized in RIPA lysis buffer (Beyotime, China) supplemented with protease and phosphatase inhibitors and centrifuged at 12,000*g* for 15 min at 4°C. The protein content in the supernatants was determined using a BCA protein assay kit (Beyotime, China).

Equal amounts of total protein (30–50 μg) were separated by SDS–PAGE and transferred onto PVDF membranes (0.45 μm; Millipore, USA). After blocking with 5% non‐fat milk for 1 h at room temperature, the membranes were incubated overnight at 4°C with the following primary antibodies: AdipoR1 (ab70362, Abcam, 1:1000), AdipoR2 (14361‐1‐AP, Proteintech, 1:1000), AURKA (66757‐1‐Ig, Proteintech, 1:1000), FOXM1 (13147‐1‐AP, Proteintech, 1:1000), and GAPDH (60004‐1‐Ig, Proteintech, 1:50000) as a loading control.

After washing, membranes were incubated with HRP‐conjugated secondary antibodies (CST91196 or CST7074, CST, 1:3000) for 1 h at room temperature. Immunoreactive bands were visualized using enhanced chemiluminescence (ECL, EpiZyme, China), and band intensities were quantified using ImageJ software.

### 2.8 Statistical Analysis

All experiments were performed independently at least three times. Data analysis was carried out using GraphPad Prism 8.0. For comparisons between two groups, an independent samples t‐test was applied to normally distributed data, while one‐way ANOVA was used to assess differences across multiple groups. Statistical significance was defined as *p* < 0.05.

## 3 Results

### 3.1 APN Promotes Apoptosis and Suppresses Proliferation of HaCaT Cells

**Figure 2:**
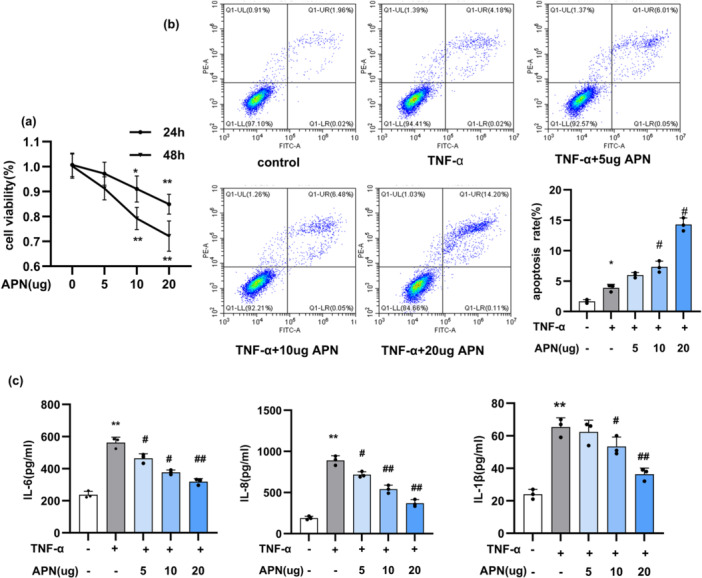
APN inhibited proliferation of HaCaT cells and reduces the inflammatory reaction induced by TNF‐α. (a) Cell viability following various APN concentrations was assessed using CCK‐8; (b) Cell apoptosis was detected using flow cytometry; (c) ELISA kits were used to measure the levels of inflammatory markers IL‐6, IL‐8, and IL‐1β in a quantitative manner. Statistical significance was determined by one‐way ANOVA. * and **: *p* < 0.05 and *p* < 0.01 when comparing to control group. #*p* < 0.05 when comparing to TNF‐α group. APN, adiponectin.

To investigate the effects of APN on HaCaT cells, we examined both cell proliferation and apoptosis. Using the CCK‐8 assay, we found that APN treatment significantly inhibited HaCaT cell proliferation in a time‐ and dose‐dependent manner (*p* < 0.05, Figure 2a). Flow cytometry analysis showed that APN therapy markedly increased the apoptosis rate of HaCaT cells, with a significantly higher percentage of apoptotic cells in the APN‐treated group compared to the control group (*p* < 0.05, Figure 2b). Collectively, these findings deonstrate that APN effectively promotes apoptosis and suppresses abnormal proliferation in HaCaT cells.

### 3.2 APN Suppresses Inflammation Response in TNF‐α‐Induced HaCaT Cells

This study evaluated the role of APN in modulating the inflammatory response in HaCaT cells induced by TNF‐α. ELISA revealed that the levels of cytokines, including IL‐8, IL‐ 6, and IL‐1β, was significantly increased in HaCaT cells following 24‐h stimulation with TNF‐α. Treatment with APN in a dose‐dependent manner significantly reduced inflammatory cytokines (*p* < 0.01, Figure 2c). These fingdings indicate that APN attenuates the inflammatory response induced by TNF‐α in HaCaT cells.

### 3.3 APN has Different Influence on AdipoR1/AdipoR2/T‐cad

**Figure 3:**
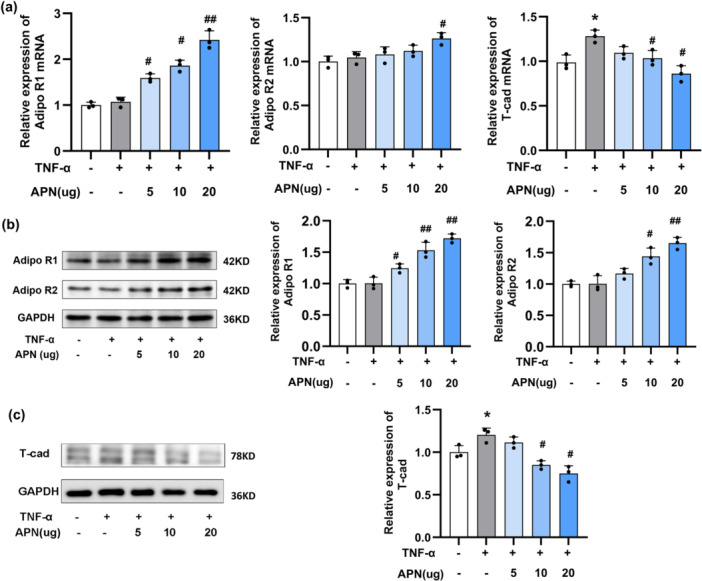
APN regulated the expression of receptor in TNF‐α‐induced HaCaT cells. (a) The mRNA expression of AdipoR1, AdipoR2 and T‐cad was detected by RT‐qPCR; (b) AdipoR1 and AdipoR2 were detected by Western blotting; (c) T‐cad expression was detected by Western blotting. Statistical significance was determined by one‐way ANOVA. *p < 0.05 when comparing to control group. # and ##: *p* < 0.05 and *p* < 0.01 when comparing with TNF‐α group. APN, adiponectin.

To explore the regulatory effects of APN on AdipoR1, AdipoR2, and T‐cad in HaCaT cells, we evaluated the protein and mRNA expression of these key molecules. RT‐qPCR analysis showed that, in a dose‐dependent manner, APN significantly upregulated the mRNA level of AdipoR1. At higher doses, APN also modestly increased the mRNA level of AdipoR2 (*p* < 0.05, Figure 3a). Consistently, Western blot analysis confirmed that APN exerted similar regulatory effects at the protein level, with AdipoR1 and AdipoR2 expression showing trends consistent with their mRNA changes(Figure 3b).

We next examined the effects of TNF‐α on T‐cad expression and assessed the potential regulatory role of APN. TNF‐α stimulation significantly increased T‐cad expression at both the protein and mRNA levels. Notably, APN treatment significantly inhibited the TNF‐α‐induced increase in T‐cad expression (*p* < 0.05, Figure 3a,c).

These findings indicate that APN upregulates both the mRNA and protein expression of AdipoR1, and to a lesser extent AdipoR2, while simultaneously suppressing TNF‑α‑induced T‑cad expression at both the transcriptional and protein levels.

### 3.4 APN Suppresses Inflammation Response by Targeting AURKA

**Figure 4:**
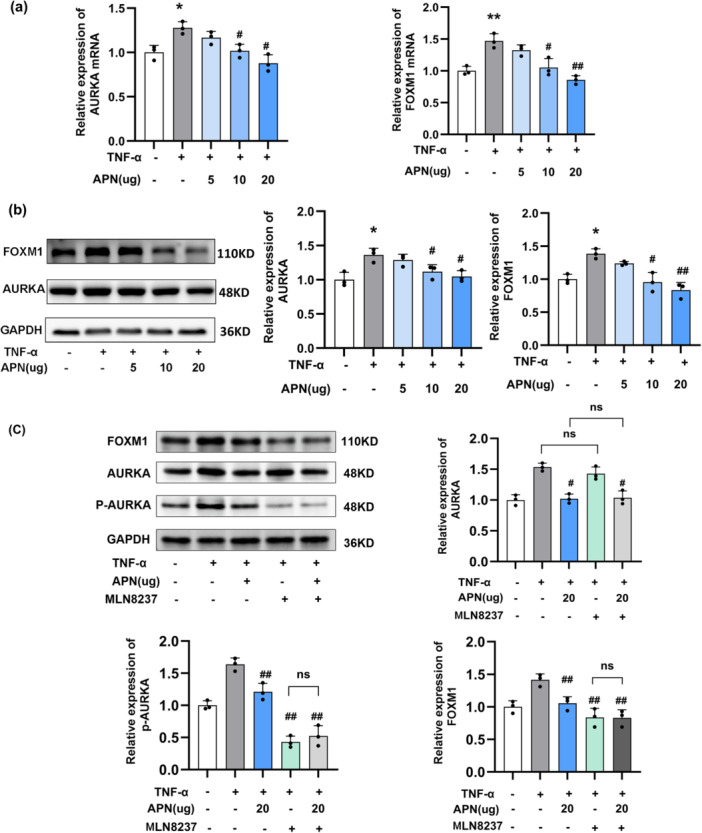
APN inhibits proliferation of TNF‐α‐stimulated HaCaT cells by targeting AURKA. RT‐qPCR and WB were used to measure the mRNA (a) and protein (b) levels of AURKA and FOXM1 in response to TNF‐α and APN (c) WB was used to assess FOXM1 expression after treatment with the AURKA inhibitor MLN8237. Statistical significance was determined by one‐way ANOVA.* and **: *p* < 0.05 and *p* < 0.01 when comparing to control group. # and ##: *p* < 0.05 and *p* < 0.01when comparing with TNF‐α group. APN, adiponectin.

For assessing the impact of APN on the expression of AURKA and FOXM1 in HaCaT cells, RT‐qPCR and WB were performed. The result revealed that, compared with the control group, TNF‐α stimulation significantly upregulated AURKA and FOXM1 expression at both protein and mRNA levels (*p* < 0.05, Figure 4a,b). This increase was observed in both mRNA and protein expression. However, after treatment with APN, the TNF‐α‐induced elevation of AURKA and FOXM1 was significantly reversed, with expression levels reduced to those comparable to the control group (*p* < 0.05, Figure 4a,b).

To further investigate the regulatory mechanism, we first confirmed that APN not only suppressed total AURKA expression (*p* < 0.05, Figure 4c) but also reduced the phosphorylation level of AURKA (p‑AURKA) (*p* < 0.01, Figure 4c). We then examined the role of MLN8237, a specific AURKA inhibitor, in the modulation of p‐AURKA and FOXM1 expression. Treatment with MLN8237 (0.2 µM) significantly suppressed p‐AURKA expression (*p* < 0.01, Figure 4c). Importantly, FOXM1 expression, which was elevated by TNF‐α in HaCaT cells, was also significantly reduced by MLN8237 (*p* < 0.05, Figure 4c). Notably, when MLN8237 was combined with APN treatment, the reduction in p‐AURKA and FOXM1 expression was more pronounced compared to either MLN8237 or APN alone, suggesting that both may regulate AURKA and its downstream target FOXM1 through similar mechanisms (Figure 4c).

Collectively, these results indicate that APN may regulate TNF‐α‐stimulated inflammation and proliferation in HaCaT cells by inhibiting AURKA, which in turn reduces the expression of FOXM1.

### 3.5 APN Alleviated IMQ‐Induced Psoriatic Dermatitis in Mice

**Figure 5:**
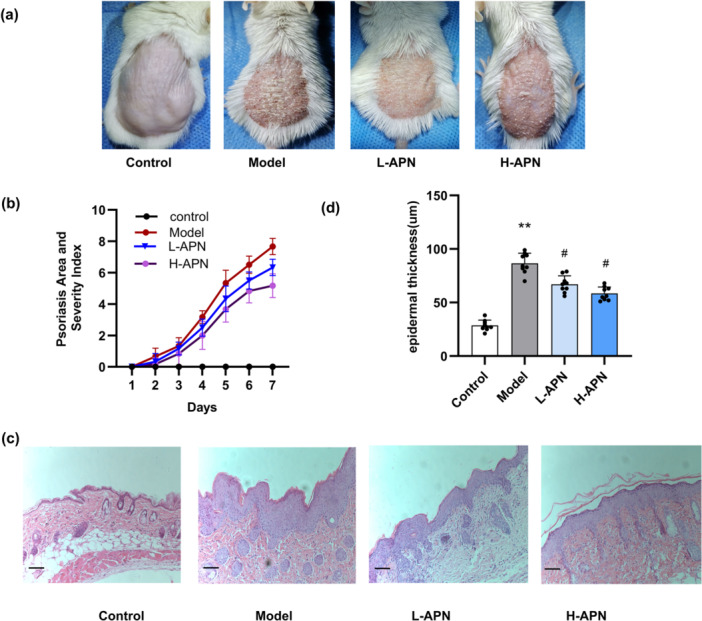
APN improved IMQ‐induced psoriatic dermatitis in mice. (a) Images of mouse back skin treated with or without APN. (b) The severity of the IMQ‐induced dorsal psoriatic dermatitis was assessed by PASI scores. (c) Representative pictures showing H&E staining at 100x magnification with a scale bar of 50 μm were captured; (d) epidermal thickness of IMQ‐induced psoriatic lesions in each group. *n* = 8 per group. Statistical analysis: (a) was analyzed using two‐way ANOVA; (b) and (d) were analyzed using one‐way ANOVA. * and **: *p* < 0.05 and *p* < 0.01 when comparing to control group. # and ##: *p* < 0.05 and *p* < 0.01when comparing with model group. l‐APN, low‐dose adiponectin; H‐APN, high‐dose adiponectin.

To investigate whether APN alleviated psoriatic dermatitis in mice, we employed IMQ‐induced psoriatic dermatitis model with and without APN therapy. The IMQ‐induced mice developed obvious psoriasis‐like dermatitis on their backs characterized by thickening, scaling and erythema (Figure 5a). Compared to the IMQ group, the administration of APN significantly reduced the severity of the psoriatic dermatitis which demonstrated by the PASI scores on days 6 and 7 (*p* < 0.05, Figure 5b).

Histological analysis revealed that IMQ‐induced psoriatic dermatitis, as shown by the downward epidermal extension, development of thickening of the acanthosis cell layer and epidermal parakeratosis. In contrast, APN treatment significantly reduced epidermal thickness and improved the skin lesion (Figure 5c). No statistically significant differences existed in PASI scores or epidermal thickness between the low‐ and high‐ dose APN groups (*p* > 0.05).

**Figure 6:**
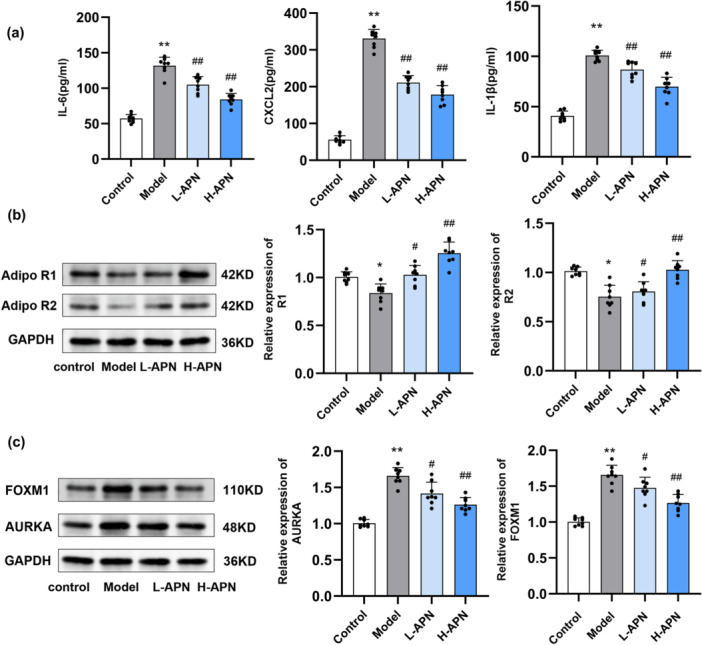
APN inhibits inflammatory cytokine production, enhances AdipoR1 and AdipoR2 expression, and suppresses AURKA–FOXM1 signaling in IMQ‐induced psoriatic mouse skin. (a) The levels of pro‐inflammatory cytokines IL‐6, CXCL2, and IL‐1β in mouse skin tissues were assessed by ELISA; (b) The expression of AdipoR1 and AdipoR2 in mouse skin was evaluated by Western blot; (c) The protein expression of AURKA and FOXM1 in mouse skin was measured by Western blot. *n* = 8 per group. Statistical significance was determined by one‐way ANOVA.* and **: *p* < 0.05 and *p* < 0.01 compared to control group. # and ##: *p* < 0.05 and *p* < 0.01 compared to IMQ model group. L‐APN, low‐dose adiponectin; H‐APN, high‐dose adiponectin.

To further assess local inflammation, we measured pro‐inflammatory cytokines in the skin tissues by ELISA. IMQ application markedly increased the levels of IL‐1β, CXCL2, and IL‐6 compared to the control group. Notably, APN treatment significantly attenuated the elevated levels of these cytokines (*p* < 0.05, Figure 6a), indicating a potent anti‐inflammatory effect of APN in vivo.

WB analysis was further conducted to examine the expression of adiponectin receptors in skin tissues. The results showed that APN treatment upregulated the expression of AdipoR1 and AdipoR2 at the protein level in IMQ‐treated mice (Figure 6b). This suggests that exogenous APN may enhance its signaling capacity via upregulation of its own receptors under inflammatory conditions.

Moreover, to validate whether the AURKA–FOXM1 axis was involved in the in vivo protective effect of APN, we analyzed their expression by WB. As expected, IMQ exposure significantly upregulated the expression of AURKA and FOXM1 in dorsal skin tissues, whereas APN administration significantly reduced their levels (*p* < 0.05, Figure 6c). These findings indicate that the AURKA–FOXM1 signaling pathway might be a crucial downstream target through which APN exerts its anti‐inflammatory and anti‐proliferative effects in the psoriatic microenvironment.

## 4 Discussion

Psoriasis, a recurrent inflammatory systemic disease, is influenced by metabolic disorders, which serve as an independent risk factor and are linked to increased disease severity [26]. Adipose tissue is currently acknowledged as a dynamic endocrine and immune organ, capable of producing a variety of bioactive molecules, which is increasingly being recognized by epidemiological studies [27]. In this study, we demonstrated that APN inhibited keratinocyte hyperproliferation and TNF‐α‐induced inflammatory cytokine secretion in a dose‐dependent manner. These results support the potential of APN as a therapy for psoriasis.

APN's biological effects on various cells, including adipocytes and macrophages, induced by TNF‐α have been studied, such as in insulin resistance, atherosclerosis, or liver disease [28, 29, 30]. However, the impact of adiponectin on keratinocytes in psoriasis remains limited. Therefore, we first confirmed the impacts of APN on apoptosis and proliferation of HaCaT cells, finding that APN effectively promotes apoptosis of HaCaT cells and inhibits abnormal proliferation. Secondly, given the pivotal role of TNF‐α in both psoriasis and psoriatic arthritis, where it induces inflammatory responses in keratinocytes [31, 32, 33], we used TNF‐α‐stimulated HaCaT cells as a cellular model for psoriasis and found that APN attenuated the TNF‐α‐stimulated inflammatory response in HaCaT cells. Moreover, in the IMQ‐induced psoriatic mouse model, APN also exhibited potent anti‐inflammatory effects, significantly reducing the levels of pro‐inflammatory cytokines such as IL‐1β, IL‐6, and CXCL2 in skin tissues, further supporting its in vivo anti‐inflammatory potential.

The anti‐inflammatory and anti‐proliferative actions of APN are facilitated through its binding to specific receptors, including AdipoR1, AdipoR2, and T‐cad. However, globular adiponectin (gAd), a biologically active form of APN, cannot bind to T‐cad, suggesting that AdipoR1 and AdipoR2 may play a more prominent role in mediating its biological functions. We observed that APN treatment reduced the mRNA and protein levels of T‐cad while simultaneously upregulating AdipoR1 and AdipoR2 expression in this study. The downregulation of T‐cad may affect AdipoR1 and AdipoR2 signaling and the anti‐inflammatory impact of APN, which need further research. Notably, in the IMQ‐induced psoriatic mouse model, the expression of AdipoR1 and AdipoR2 was significantly decreased compared to control mice, APN treatment effectively restored the expression levels of both receptors, suggesting that under inflammatory conditions, APN may enhance its signaling efficacy by upregulating its own receptors in vivo.

Most importantly, our study discovered the involvement of AURKA and FOXM1 in proliferation and TNF‐α‐induced inflammation of keratinocyte, as well as the regulatory role of APN. In the past, AURKA and FOXM1 were both recognized as pro‐oncogenes, and their relevance to psoriasis has been increasingly noted [21, 22, 23]. AURKA is known to be upregulated in psoriatic lesions and plays a role in promoting keratinocyte proliferation and inflammasome activation [23, 24, 25]. Consistent with these studies, the present study showed that TNF‐α stimulation notably enhanced the mRNA and protein expression of AURKA and FOXM1 in HaCaT cells. Notably, treatment with APN markedly attenuated the TNF‐α‐induced upregulation of AURKA and FOXM1, suggesting that APN may exert its anti‐proliferative effects by inhibiting AURKA, thereby downregulating FOXM1 expression.

Further support for this mechanism was provided by the use of the AURKA inhibitor MLN8237, which significantly reduced the expression of p‐AURKA and simultaneously suppressed FOXM1 expression in HaCaT cells, reinforcing the hypothesis that AURKA serves as an upstream regulator of FOXM1 in keratinocytes. Interestingly, when MLN8237 was combined with APN, no further suppression of p‐AURKA or FOXM1 was observed compared to either treatment alone, implying that APN and MLN8237 may regulate this pathway through overlapping or convergent mechanisms.

Importantly, the results from the IMQ‐induced psoriatic mouse model mirrored the in vitro findings. IMQ treatment led to significant upregulation of AURKA and FOXM1 protein levels in mouse skin, which were markedly reduced following APN administration. This in vivo validation reinforces the hypothesis that the AURKA–FOXM1 signaling pathway is a critical downstream target through which APN exerts its anti‐psoriatic effects, highlighting its potential for therapeutic exploitation in psoriasis.

Finally, we evaluated the impact of APN on IMQ‐stimulated psoriatic dermatitis in mice. Treatment with APN effectively alleviated psoriatic lesions; however, no statistical differences were observed in PASI scores or epidermal thickness between the low‐dose and high‐dose APN groups. This suggests that further increases in APN concentration do not provide additional therapeutic benefits. A plausible explanation for this phenomenon is the saturation of APN receptor signaling pathways. Once receptor occupancy reaches its maximum capacity, further increases in APN concentration are unlikely to enhance downstream signaling or amplify its therapeutic effects.

### 4.1 Limitations of the Present Study

This study has several limitations that should be taken into account when interpreting the results. First, although the anti‐inflammatory and anti‐proliferative effects of adiponectin (APN) were demonstrated in vitro using HaCaT cells and in vivo using an IMQ‐induced psoriatic mouse model, the specific molecular mechanisms of APN in human psoriatic lesions remain unclear. Second, while the study explored the effects of APN on AURKA and FOXM1, other potential signaling pathways regulated by APN were not investigated. Additionally, only a single keratinocyte cell line was used in this study, which may not fully represent the complex interactions in human skin. Future studies should focus on validating these findings in human patients and exploring other potential molecular targets.

## 5 Conclusion

In conclusion, APN effectively attenuated the hyperproliferation of HaCaT cells, reduced TNF‐α‐stimulated inflammation, and ameliorated psoriatic dermatitis in mouse model. The APN's antiproliferative and anti‐ inflammatory properties on HaCaT cells were associated with the downregulation of AURKA, a key regulator of cell division and inflammation. Notably, AURKA was found to modulate FOXM1 expression, demonstrating the vital role of the AURKA‐FOXM1 axis in psoriasis pathophysiology. These results suggest that APN holds potential as an anti‐psoriatic agent and underscore the therapeutic value of targeting AURKA and FOXM1 for the gene‐targeted therapies in psoriasis treatment.

## Author Contributions

Lingling Zhang designed the study, performed the experiments, and prepared the article. Chunxi Ke and Feng Shi was involved in preparing the initial draft of the article. Yun Shen conducted data processing and contributed to the analysis. Qingqing Jiao contributed to the study design, provided guidance on the experimental approach, and was involved in proofreading the article. Jiang Ji was the overall study coordinator, supervised the entire research process, and was involved in article preparation and final proofreading. All authors read and approved the final version of the article.

## Ethics Statement

The Ethics Committee of Gongli Hospital, Shanghai Pudong New Area, granted approval for this study (Grant No. GLYY1s2025‐012).

## Conflicts of Interest

The authors declare no conflicts of interest.

## Data Availability

Data available on request from the authors.
